# Temporal and Spatial Nearest Neighbor Values Based Missing Data Imputation in Wireless Sensor Networks

**DOI:** 10.3390/s21051782

**Published:** 2021-03-04

**Authors:** Yulong Deng, Chong Han, Jian Guo, Lijuan Sun

**Affiliations:** 1 College of Computer, Nanjing University of Posts and Telecommunications, Nanjing 210003, China; dyl@njupt.edu.cn (Y.D.); hc@njupt.edu.cn (C.H.); guoj@njupt.edu.cn (J.G.); 2 Jiangsu High Technology Research Key Laboratory for Wireless Sensor Networks, Nanjing University of Posts and Telecommunications, Nanjing 210003, China

**Keywords:** wireless sensor networks, missing data, imputation, temporal and spatial nearest neighbor values, regression

## Abstract

Data missing is a common problem in wireless sensor networks. Currently, to ensure the performance of data processing, making imputation for the missing data is the most common method before getting into sensor data analysis. In this paper, the temporal and spatial nearest neighbor values-based missing data imputation (TSNN), a new imputation based on the temporal and spatial nearest neighbor values has been presented. First, four nearest neighbor values have been defined from the perspective of space and time dimensions as well as the geometrical and data distances, which are the bases of the algorithm that help to exploit the correlations among sensor data on the nodes with the regression tool. Next, the algorithm has been elaborated as well as two parameters, the best number of neighbors and spatial–temporal coefficient. Finally, the algorithm has been tested on an indoor and an outdoor wireless sensor network, and the result shows that TSNN is able to improve the accuracy of imputation and increase the number of cases that can be imputed effectively.

## 1. Introduction

The dataset will become incomplete if some data are lost in the acquisition stage, which may be caused by many different reasons [1]. Generally, most of them are caused by the human errors especially when the data come from manual questionnaires or by the non-human errors when the data are obtained from a system automatically, for example, the faults of the sensors or communication units in wireless sensor networks. The latter will be focused on in this paper. However, most of the theories of data processing and related algorithms are founded on complete datasets so far. Missing data in the dataset which make the dataset incomplete will have an impact on the subsequent data processing stage, for instance, degrading the quality of classification, even making the processing task fail. For example, in human activity recognition where the Support Vector Machine (SVM) is applied as the classification tool. Two per cent of missing data will cause the recognition rate to decrease to 80%. When the missing rate increases to 5%, the recognition rate will drop down to 65%, and if the missing rate is over 10%, the recognition rate will be below 50%, which may make it unavailable in the recognition task [2]. In a very few of situations, we can get the complete dataset by simply discarding parts of it which contain the missing data, but in most of cases, the missing data can be imputed by the estimation values, and a complete dataset will be obtained before we start the data processing in the next step; therefore, it is important to improve the performance of missing data imputation. 

Currently, wireless sensor networks (WSNs) contribute considerable data of the physical environment and events through sensors on the deployed nodes which are connected with a communication network. Generally, in WSNs, data are acquired by nodes and sent to the processing center, the unstable and even dramatic change of environment where the WSNs deployed may increase the chance of sensor faults, and the capacity fade of their power system that is commonly composed of batteries will cause the malfunction of the nodes. This work principle of WSN increases the possibility to lose data during the data acquisition. That means the dataset obtained from WSN will be an incomplete dataset in a high likelihood. Among all methods to deal with the incomplete WSN dataset, one of them is to work on it directly by special algorithms without imputation. Some researchers design effective processing methods on their datasets. Bryan Conroy [3] proposed the Dynamic–Abstain–Boost. It is a dynamic classifier obtained from a two-stage machine learning algorithm, in which the low-dimensional classifiers are combined in the ensemble learning stage. The classifier works directly on the incomplete dataset and is tested on the intensive care unit (ICU) clinical dataset which consists of huge number of physiological sampled measurement data, where some of them are missing because of insufficient monitoring. Vladimir Gorodetskytichu [4] developed a lattice based direct mining method on the binary dataset with missing values. The approach is applied to extract the classification rules, based on upper and low bounds of the rules’ sets. After testing on a classification criterion, it will select a subset of rules, which will be used for classification. The mining algorithm has been applied on raw data streams in the computer network. These previous work shows the possibility to process incomplete datasets without imputation. However, they are limited to designated data processing on special application scenarios whereas most of data processing tasks in WSNs still require the complete datasets, which fuels the demand for research on the imputation algorithms in WSNs.

The imputation methods are closely connected with the causes of the data missing and the characteristics of the incomplete datasets. There are lots of different reasons that contribute to the data missing in WSNs, such as the failures of the sensors on nodes, the abnormalities of the wireless communication or the malfunction of the power supply, for instance, the capacity fade of the battery may lead to the abnormalities of the measurements from sensors [5]. A complete dataset in WSNs should have measurement values on all nodes at all time points in an observed time window; if not, we can detect there is missing data in the test case, and the dataset is incomplete. Missingness mechanisms [1] are an important feature of the incomplete dataset; missing completely at random (MCAR) occurs when the probability of a case having a missing value for an observed attribute is independent of either the known values or the missing data, which is a typical situation when the data loss occurs in WSNs and accounts for large proportions of the cases with missing values. Therefore, most of the current researchers use MCAR for the theoretical study and experiments [6]; likewise, in this paper we carry out the study on the imputation algorithms for cases in the MCAR mechanism.

At present, machine learning techniques are the main tools for researchers to design imputation techniques of missing values in WSNs, which are applied to find features of the dataset, exploit the information in the non-missing data and figure out estimators for the missing values. Different research studies have been carried out on this area. In [7], Roman Tkachenko proposed the GRNN-SGTM. General regression neural networks have been used in the ensemble algorithm. Two successively connected general regression neural networks (GRNN) and a successive geometric transformation model (SGTM) neural-like structure are assembled. In addition, for improving the accuracy of estimation of missing values, a weighted summation is applied in the algorithm. Nan Jiang applied the association rule mining techniques in the closed item sets based association rule mining (CARM) [8], which is applied to extract the most recent association rules between sensors and work out the missing values of sensors in a traffic network.

It is worth noting that the data analysis proves that there are strong correlations among data in the dimensions of time and space in WSNs. Regression, as one of the classical machine learning algorithms, can exploit the correlation effectively and make the processing procedure simpler as well, which is conducive to reduce the expense in the process. In [9], Liqiang Pan introduced an adaptive missing data estimation algorithm (CIAM). It is a self-adaptive algorithm in which a multiple regression model is applied to make the imputation based on the spatial correlation among sensor nodes and improve the accuracy of the missing value estimation. Another imputation method, a new estimation model based on a spatial temporal correlation analysis (STCAM), is presented by Xiaojun Ren [10], for obtaining better estimates of the missing value, four sub-algorithms based on regression are combined to exploit the spatial and temporal correlation in the datasets of an indoor sensor network and a traffic network. 

In this paper, we focus on the research route to make imputation based on the spatial and temporal correlation and introduce a new imputation algorithm for missing values in WSNs, the temporal and spatial nearest neighbor values based missing data imputation (referred to as TSNN). Similar to the previous research work, regression tool is applied in our algorithm but in a new way to figure out the estimates based on the temporal and spatial nearest neighbor values which are defined on the bases of geometrical distance and the data distance. Through this approach, the correlation hidden in the data can be exploited more effectively, and the power of regression can be boosted to improve both the percentage of cases in which a missing value can be estimated and the accuracy of the estimation for missing values.

The rest of this paper is organized as follows. First, in Section 2, we make a review of typical imputation methods in WSNs and present the main contributions of our work. Then, in Section 3, the main reasons of data loss and the scenarios of data missing in WSNs have been described. After that, we give definitions of four kinds of nearest neighbor values from the perspective of time and space dimensions and analyze the correlations between these values and the raw value. Based on them, we elaborate our new imputation algorithm TSNN. Next, in Section 4, we describe the experiments and the results for our method. Finally, we make a discussion and conclude our research work in Section 5 and Section 6.

## 2. Related Work

It is an effective way to make imputation for the missing values in WSNs from perspective of time and space dimensions. Many research studies have been carried out on this field. We have summarized representative methods proposed by previous researchers as follows:

- Linear interpolation model (LIN) Algorithm [11].

The temporal correlation among the measurements of a sensor on a node $s_{i}$ is utilized in this algorithm. It applies the linear interpolation to figure out the estimated value $\hat{t\left(s_{i},u_{miss}\right)\;}$ based on two time points $u^{\prime }$ and $u^{\prime \prime }$ that are nearest to the time point $u_{miss}\;$ at which the measurement is missed inside the time window W.

(1) $$\hat{t\left(s_{i},u_{miss}\right)\;}=\;t\left(s_{i},u^{\prime \prime }\;\right)+\;\frac{\left(t\left(s_{i},u^{\prime }\;\right)-\;t\left(s_{i},u^{\prime \prime }\;\right)\right)\left(u_{miss}-\;u^{\prime \prime }\right)}{u^{\prime }-u^{\prime \prime }}$$

where $u^{\prime }$, $u^{\prime \prime }\in W$.

- K-nearest temporal neighbors (TKNN) Algorithm.

TKNN is a special version of the nearest neighbors method (NNs) [12], in which the neighbor values come from the nearest time points. When there is more than one sensor on a same node $s_{i}$, the algorithm applies the measurements of the sensor without missing values to find K temporal nearest neighbors $I\left(i,j\right)$ inside the time window W, meanwhile, the weight coefficient $W_{td}$ will contribute to the final calculation of the estimated value $\hat{t\left(s_{i},u_{miss}\right)\;}$.

(2) $$\hat{t\left(s_{i},u_{miss}\right)\;}=\underset{I\left(i,j\right)}{\sum }W_{td}\left(u_{j},u_{q}\right)t\left(s_{i},u_{q}\right)\;$$

where $u_{q}\in I\left(i,j\right)$.

- K-nearest spatial neighbors (SKNN) Algorithm,

Similar to TKNN, the algorithm is another version of NNs, in which the neighbor values come from the nearest spatial neighbors. It is based on the premise that there is more than one sensor on the same node $s_{i}$. Instead of temporal nearest neighbors, K spatial nearest neighbors $G\left(i,j\right)\;$ inside the time window W will be found, and their measurements are used to figure out the estimated value $\hat{t\left(s_{i},u_{miss}\right)}$, combined with the weight coefficient $W_{sd}$. In [13], the algorithm is applied to impute the missing value of gene in an experiment where K non-missing values of the deoxyribonucleic acid (DNA) in other experiments are selected as spatial nearest neighbors.

(3) $$\hat{t\left(s_{i},u_{miss}\right)\;}=\underset{G\left(i,j\right)}{\sum }W_{sd}\left(s_{i},g\left(i,j\right)\right)t\left(g\left(i,j\right),u_{j}\right)\;$$

where $\;g\left(i,j\right)\in G\left(i,j\right)$.

- Applying K-nearest neighbor estimation (AKE) Algorithm [14].

Assumed that there exist spatial nearest neighbors $G\left(i,j\right)\;$ of the sensor on a node $s_{i}$ which loses data inside the time window W, the correlation can be find among the measurements of the node $s_{i}$ and its spatial nearest neighbors on the time points without missing values; the algorithm uses them to calculate the estimated value $\hat{t\left(s_{i},u_{miss}\right)\;}$ by linear regression, where the weight coefficient is applied to figure out the contributions of the different spatial nearest neighbors.

(4) $$\hat{t\left(s_{i},u_{miss}\right)\;}=\underset{G\left(i,j\right)}{\sum }W_{k}\hat{t\left(s_{i},u_{miss}\right)\;\left(k\right)}$$

where

(5) $$\hat{t\left(s_{i},u_{miss}\right)\;\left(k\right)}=\alpha_{s_{i}}\left(k\right)+\beta_{s_{i}}\left(k\right)t\left(g\left(i,j\right),u_{miss}\right)$$

(6) $$W_{k}=\frac{R_{si}^{2}\left(k\right)}{\sum_{G\left(i,j\right)}R_{s_{i}}{}^{2}\left(g\left(i,j\right)\right)}$$

(7) $$R_{si}^{2}\left(k\right)\;=\frac{\sum_{U_{sample}}{\left(\hat{t\left(s_{i},u_{sample}\right)\left(k\right)\;}-\bar{t\left(s_{i},u_{sample}\right)\left(k\right)}\;\right)}^{2}}{\sum_{U_{sample}}{\left(t\left(s_{i},u_{sample}\right)\left(k\right)\;-\bar{t\left(s_{i},u_{sample}\right)\left(k\right)}\;\right)}^{2}}$$

where $g\left(i,j\right)\in G\left(i,j\right)$, $\alpha_{s_{i}}\left(k\right)$ and $\beta_{s_{i}}\left(k\right)$ are regression coefficients. $W_{k}$ is the weight that is applied to calculate the estimated value, in which, $R_{si}^{2}\left(k\right)$ is the percentage of variation explained by the regression model.

- Data estimation using statistical model (DESM) Algorithm [15].

While there are both temporal nearest neighbors $I\left(i,j\right)$ and spatial nearest neighbors $G\left(i,j\right)\;$ of the sensor on a node $s_{i},$ which loses data inside the time window W, both the temporal and spatial correlations can be used in the algorithm. Therefore, the estimated value $\hat{t\left(s_{i},u_{miss}\right)\;}$ will be figured out based on the measurements of temporal nearest neighbors and spatial nearest neighbors. A correlation coefficient is obtained from the node $s_{i},$ and its spatial neighbors inside the time window, which will be used as the weight coefficient to evaluate the contribution of them.

(8) $$\hat{t\left(s_{i},u_{miss}\right)}=\;\left(1-\alpha \right)t\left(s_{i},u^{\prime }\right)+\alpha t\left(s_{i},u_{j}\right)\left(1+\frac{t\left(g\left(i,u_{miss}\right),u_{miss}\right)-t\left(g\left(i,u_{miss}\right),u_{j}\right)}{t\left(g\left(i,u_{miss}\right),u_{j}\right)}\right)$$

where $u^{\prime }\in W,\;\;g\left(i,j\right)\in G\left(i,j\right)$ and weight parameter α can be computed as:

(9) $$\alpha =\frac{Cov(T\left(s_{i}\right),T\left(g\left(i\right)\right)}{\sigma T\left(s_{i}\right)\sigma T\left(g\left(i\right)\right)}$$

Methods discussed in the above research work represent the typical ways to utilize the information hidden in the WSNs data from the perspective of time and space dimensions to make estimates for the missing values; they are effective, but there are still some deficiencies and further work is required to correct them.

- The temporal and spatial correlations are considered separately or taken into account simultaneously in the research work. LIN and TKNN only make use of data in time dimension while SKNN and AKE utilize data in space dimension. DESM combines both of the time and space information to make imputation and balances them depending on a simple correlation coefficient. However, the way to fully exploit the time and space information is expected in order to improve the performance of the imputation;
- Most of research works focus on improving the accuracy of the imputation, but few of them consider the way to improve the percentage of cases in which a mission value can be estimated;
- Little research has been conducted to study the scenarios in which more than one sensor on the node has lost data during their work.

In this paper, we do research by addressing the problems above and propose a new imputation algorithm TSNN. The main contributions of this work are described as follows:

- Temporal and spatial nearest neighbor values are defined from two perspectives: geometrical distance and data distance, which make it available to exploit the temporal and spatial correlation hidden in the data more fully. In this situation, as the tool applied in the algorithm, linear regression shows better performance to make estimates for missing values, which contributes to better accuracy of imputation;
- Four temporal and spatial nearest neighbor values are combined into the algorithm, which makes it flexible to adapt to different situations in which parts of the temporal or spatial information may be unavailable. Benefiting from it, the percentage of cases in which a mission value is improved;
- Three scenarios in which there is more than one sensor on the WSNs node have been considered in the algorithm, which are consistent with actual conditions when the data get lost on the sensors;
- For evaluating the algorithm more reasonably, different from other research work, the raw datasets are utilized directly in most of our experiments without the preprocessing steps such as the mean imputation. Meanwhile, in an indoor and an outdoor WSNs datasets, the nodes have been chosen randomly in the experiments and the performance is evaluated on both sides: the percentage of cases in which a mission value can be estimated and the accuracy of the estimation for missing values.

## 3. Materials and Methods

### 3.1. Missing Data in WSNs

In WSNs, the data source are the nodes on which the sensors provide measurements. In this paper, we only discuss the centralized data processing WSNs, namely, all data coming from the nodes will be sent by a wireless communication network to the processing center of the WSN, where the data can be stored and processed. Usually, the processing center is a PC or server that has large computing power and enough memory for dealing with the data sent from nodes effectively. For collecting different physical data in the environment, generally, there is more than one sensor on the node. In most cases, each sensor is responsible for acquiring a type of physical quantity, and if it fails, the corresponding measurement value will be missing. One way to improve reliability is to equip the node with two or more same sensors for obtaining the same quantity, but it is beyond the scope of this paper. A typical example which has nodes equipped with multiple sensors is the WSN deployed in the Intel Berkeley Research lab [16], each node has a weather board equipped with four Mica2Dot sensors, collecting humidity, temperature, light and voltage values at the location of the nodes every 31 s. The measurements are all physical quantities of the environment except for voltage value. In this paper, only temperature and humidity data are used. In addition, the actual reliabilities of the sensors may be different, and they are affected by the devices or the circuits. Therefore, for simplicity in the later discussion, the odds that data missing due to the fault of sensors are assumed to be able to preset in different scenarios. In actual WSNs, there are three main reasons the data are lost on the node. Suppose there are two sensors on a node for collecting temperature and humidity.

- The communication unit on the node fails at some time points, and the data cannot be sent to the data processing center, so both measurements of the sensors, the temperature and the humidity data will be missing at these time points;
- The communication unit on the node works properly, but due to the failure of the data processing center or the fault in the data transmission, the data coming from the node cannot be received by the data processing center; likewise, the temperature and the humidity data will be missing at this time point;
- The communication unit on the node works properly, but because of the fault of the sensors or the node itself, for instance, the capacity fade of battery, both measurements or one of them, becomes abnormal, the spike, for example [17]. This data has been sent to the data processing center but will be removed because it has been judged to be abnormal; therefore, the temperature and the humidity data or one of them will be missing at this time point.

According to the cases above, in a time window, the scenarios of data missing in a node can be classified into three types: both temperature and humidity data are missing at all time points; the temperature (humidity) data are missing, but the humidity (temperature) data are available at all time points; the temperature (humidity) data are missing, but the humidity (temperature) data are available at some of the time points in certain probability. These scenarios have been considered into our new imputation algorithm. Moreover, generally, there exist correlations among the measurements of sensors on the same node, for example, the temperature and the humidity data. For exploiting the correlative relationships, we define the nearest neighbor values from the perspective of the time and space dimensions, which can take better advantage of the correlations between sensors on the same node and on the neighboring nodes as well. For the convenience of discussion, in this paper, it is assumed that there are only two sensors on a node in the WSN, for collecting temperature and humidity data separately, and the three types of scenarios will be used in the algorithm design and experiments.

### 3.2. Temporal and Spatial Nearest Neighbor Values for a Node in WSNs

#### 3.2.1. Definition and Computation of Temporal and Spatial Nearest Neighbor Values

As the basis of our TSNN imputation algorithm, we define four types of nearest neighbor values from the perspective of time and space dimensions; they are spatial nearest neighbor value in geometrical distance (SGNN), spatial nearest neighbor value in data distance (SDNN), temporal nearest neighbor value in time distance (TTNN) and temporal nearest neighbor value in data distance (TDNN).

In a wireless sensors network, nodes often work continuously, so we are usually concerned with the physical measurement values in a time period. Given a physical space P, let $S=\left\{s_{1},s_{2},\ldots ,s_{n-1},s_{n}\right\}$ be a set of n sensor nodes, $s_{i}\in S$, and a time window W and let $W=\left\{u_{1},u_{2},\ldots ,u_{m-1},u_{m}\right\}$ be a set of m time points, $u_{j}\in W$. For each node $s_{i}$, $G\left(i,j\right)$ represents its spatial nearest neighbors at time point $u_{j},$ and the number of the spatial nearest neighbors is $k_{s}$; $I\left(i,j\right)$ represents its temporal nearest neighbors at time point $u_{j},$ and the number of the temporal nearest neighbors is $k_{t}$. The node $s_{i}$ has two sensors which are used to get temperature value and humidity value, respectively, at time point $u_{j}$ on its location in the physical space; the temperature value is denoted by $t(s_{i},u_{j\;}),$ and the humidity value is denoted by $h(s_{i},u_{j\;})$.

**Definition** **1.** *Spatial Nearest Neighbor Value in Geometrical Distance (SGNN): Let* $t_{sgnn}\left(s_{i},u_{j}\right)$ *and* $\;h_{sgnn}\left(s_{i},u_{j}\right)$ *be the nearest spatial neighbor value of node* $s_{i}\;$ *for the temperature value and the humidity value at time point* $u_{j}$ *in geometrical distance, respectively.*

The geometrical distance is calculated by the positions of the node $s_{i}$ and its spatial neighbors in space. The geometrical distance between two nodes is denoted by $\;D_{sg\;}\left(\cdot ,\cdot \right)$, and the nearest spatial neighbor value for temperature value in geometrical distance $t_{sgnn}\left(s_{i},u_{j}\right)$ can be given as:

(10) $$t_{sgnn}\left(s_{i},u_{j}\right)\;=\left\{\begin{array}{cc} t\left(f_{t}\left(i,j\right),u_{j}\right) & \;\;\;G\left(i,j\right)\;\neq \;\emptyset \\ NA & \;\;\;G\left(i,j\right)\;=\emptyset \end{array}\right.$$

Similarly, the nearest spatial neighbor value for humidity value in geometrical distance is given as:

(11) $$h_{sgnn}\left(s_{i},u_{j}\right)\;=\left\{\begin{array}{cc} h\left(f_{h}\left(i,j\right),u_{j}\right) & \;\;\;G\left(i,j\right)\;\neq \;\emptyset \\ \;NA & \;\;\;G\left(i,j\right)\;=\emptyset \end{array}\right.$$

where NA means the value is not available, and $f\left(i,j\right)$ describes the spatial nearest neighbor of node $s_{i}$ at time point $u_{j}$ in geometrical distance, which is computed as

(12) $$f_{t}\left(i,j\right)\;=\left\{g_{x}\left(i,j\right)|\begin{array}{c} \forall g_{y}\left(i,j\right),\;t\left(g_{y}\left(i,j\right),u_{j}\right)\;\neq NA;\;\exists g_{x}\left(i,j\right)\;that\;makes\\ \;D_{sg}\left(g_{x}\left(i,j\right),s_{i}\right)\leq D_{sg}\left(g_{y}\left(i,j\right),s_{i}\right),\;\;t\left(g_{x}\left(i,j\right),u_{j}\right)\;\neq NA\; \end{array}\right\}$$

(13) $$f_{h}\left(i,j\right)\;=\left\{g_{x}\left(i,j\right)|\begin{array}{c} \forall g_{y}\left(i,j\right),h\left(g_{y}\left(i,j\right),u_{j}\right)\;\neq NA;\;\exists g_{x}\left(i,j\right)\;that\;makes\;\\ D_{sg}\left(g_{x}\left(i,j\right),s_{i}\right)\leq D_{sg}\left(g_{y}\left(i,j\right),s_{i}\right),\;\;h\left(g_{x}\left(i,j\right),u_{j}\right)\;\neq NA\; \end{array}\right\}$$

where $g_{x}\left(i,j\right)\in G\left(i,j\right)$, $g_{y}\left(i,j\right)\in G\left(i,j\right)$ and x≠y.

**Definition** **2.** *Spatial Nearest Neighbor Value in Data Distance (SDNN): Let* $t_{sdnn}\left(s_{i},u_{j}\right)$ *and* $h_{sdnn}\left(s_{i},u_{j}\right)$ *be the nearest spatial neighbor values of node* $s_{i}\;$ *for the temperature value and the humidity value at time point* $u_{j}$ *in data distance, respectively.*

Different from the geometrical distance, the data distance between two nodes is calculated by the data of the nodes and is only available when there are more than one types of physical values in the data, and it indicates the relationship between different types of values acquired by nodes. The data distance between two nodes is denoted by $\;D_{sd\;}\left(\cdot ,\cdot \right)$. For example, there are two nodes and each of them has obtained three types of values, A, B and C. Values a1, b1 and c1 are from node1, and values a2, b2 and c2 are from node2. Then, to value A, the data distance between node 1 and node 2 can be calculated as $\;D_{sd\;}\left(node1,\;node2\right)=\sqrt{{\left(b1-b2\right)}^{2}+{\left(c1-c2\right)}^{2}}$. In this paper, we only consider that there are two types of values obtained on nodes, the temperature and the humidity. To temperature value, the data distance between them can be calculated by humidity values and vice versa. Therefore, to the temperature value, the data distance between nodes $s_{p}$ and $s_{q}$ at time $u_{j}$ can be computed as $\;D_{sd\;}\left(s_{p},s_{q}\right)=\sqrt{{\left(h\left(s_{p},u_{j}\right)-h\left(s_{q},u_{j}\right)\right)}^{2}}=\left|h\left(s_{p},u_{j}\right)-h\left(s_{q},u_{j}\right)\right|$; likewise, to the humidity value, the data distance between nodes $s_{p}$ and $s_{q}$ at time point $u_{j}$ can be computed as $\;D_{sd\;}\left(s_{p},s_{q}\right)=\sqrt{{\left(t\left(s_{p},u_{j}\right)-t\left(s_{q},u_{j}\right)\right)}^{2}}=\left|t\left(s_{p},u_{j}\right)-t\left(s_{q},u_{j}\right)\right|$.

Then the spatial nearest neighbor value of node $s_{i}$ for the temperature value at time point $u_{j}\;$ in data distance can be computed as

(14) $$t_{sdnn}\left(s_{i},u_{j}\right)\;=\left\{\begin{array}{cc} \underset{G\left(i,j\right)}{\sum }W_{sd}\left(s_{i},g\left(i,j\right)\right)t\left(g\left(i,j\right),u_{j}\right)\; & h\left(s_{i},u_{j}\right)\;\neq \;NA\;and\;\forall g\left(i,j\right)\;\in \;G\left(i,j\right),\exists h(g\left(i,j\right),u_{j})\;\neq \;NA\\ NA & h\left(s_{i},u_{j}\right)\;=\;NA\\ NA & \forall g\left(i,j\right)\;\in \;G\left(i,j\right),\exists h(g\left(i,j\right),u_{j})\;=\;NA \end{array}\right.$$

Similarly, the spatial nearest neighbor value of node $s_{i}$ for the humidity value at time point $u_{j}\;$ in data distance can be computed as

(15) $$h_{sdnn}\left(s_{i},u_{j}\right)\;=\left\{\begin{array}{cc} \underset{G\left(i,j\right)}{\sum }W_{sd}\left(s_{i},g\left(i,j\right)\right)h\left(g\left(i,j\right),u_{j}\right)\; & t\left(s_{i},u_{j}\right)\;\neq \;NA\;and\;\forall g\left(i,j\right)\;\in \;G\left(i,j\right),\exists t(g\left(i,j\right),u_{j})\;\neq \;NA\\ NA & t\left(s_{i},u_{j}\right)\;=\;NA\\ NA & \forall g\left(i,j\right)\;\in \;G\left(i,j\right),\exists t(g\left(i,j\right),u_{j})\;=\;NA \end{array}\right.$$

where $g\left(i,j\right)\in G\left(i,j\right)$, and the weight $W_{sd}$ can be computed as

(16) $$W_{sd}\left(s_{i},g\left(i,j\right)\right)\;=\frac{K\left(\;D_{sd\;}\left(s_{i},g\left(i,j\right)\right)\right)}{\sum_{G\left(i,j\right)}K\left(\;D_{sd\;}\left(s_{i},g\left(i,j\right)\right)\right)}$$

where $K\left(\cdot \right)$ is a kernel function, for example Gaussian.

Especially, if $h\left(s_{i},u_{j}\right)$ is missing, the $t_{sdnn}\left(s_{i},u_{j}\right)$ will be unavailable. Similarly, $h_{sdnn}\left(s_{i},u_{j}\right)$ will be unavailable when $t\left(s_{i},u_{j}\right)$ is missing; the unavailable spatial nearest neighbor value is denoted as NA.

**Definition** **3.** *Temporal Nearest Neighbor Value in Time Distance (TTNN): Let* $t_{ttnn}\left(s_{i},u_{j}\right)$ *and* $h_{ttnn}\left(s_{i},u_{j}\right)$ *be the nearest spatial neighbor values of node* $s_{i}$ *for the temperature value and the humidity value at time point* $u_{j}$ *in time distance, respectively.*

The time distance is calculated by the time point $u_{j}$ and its near time points in the time window W. The time distance between two time points is denoted by $\;D_{tg\;}\left(\cdot ,\cdot \right)$. It is worth noting that there are important differences between TTNN and SGNN. For the node $s_{i}$, SGNN comes directly from the value of one of its actual nearest spatial neighbors at the same time point. However, TTNN comes from the values of its two temporal nearest neighbors, i.e., the values of its two nearest time points on the same node; moreover, different from SGNN, these values are not applied directly; instead, they are used to calculate the final temporal nearest neighbor value by the formula presented below.

Supposed that $u^{\prime }$ and $u^{\prime \prime }$ are the two nearest time points of $u_{j}$ for node $s_{i}$ in window W. 

Given $u_{j}$, we have

(17) $$u^{\prime }=\;\left\{u_{x}|\forall u_{y}\in W,\;t\left(s_{i},u_{y}\right)\;\neq NA;\;\exists u_{x},\;t\left(s_{i},u_{x}\right)\;\neq NA\;that\;makes\;D_{tg}\left(u_{x},u_{j}\right)\leq D_{tg}\left(u_{y},u_{j}\right)\;\right\}$$

where $u_{x}\;\in W$ and  x≠y.

(18) $$u^{\prime \prime }=\;\left\{u_{x}|\forall u_{y}\in W,\;t\left(s_{i},u_{y}\right)\;\neq NA;\;\exists u_{x},\;t\left(s_{i},u_{x}\right)\;\neq NA\;that\;makes\;D_{tg}\left(u^{\prime },u_{j}\right)\leq D_{tg}\left(u_{x},u_{j}\right)\leq D_{tg}\left(u_{y},u_{j}\right)\;\right\}$$

where $u_{y}\;\in W$ and  x≠y, in which, the time distance between two time points is denoted by $\;D_{tg\;}\left(\cdot ,\cdot \right)$.

Then the temporal nearest neighbor value of node $s_{i}$ for the temperature value in time distance can be computed as

(19) tttnnsi,uj =tsi,u′u″−uj+tsi,u″uj−u′u″−u′  ∃ u′∈W,u″∈W,u′≠u″tsi, u′  ∃ u′∈WNA  ∃ u′∈W

Similarly, the temporal nearest neighbor value of node $s_{i}$ for the humidity value in time distance can be computed as

(20) httnnsi,uj =hsi,u′u″−uj+hsi,u″uj−u′u″−u′  ∃u′∈W,u″∈W,u′≠u″hsi, u′  ∃ u′∈WNA  ∃ u′∈ W

**Definition** **4.** *Temporal Nearest Neighbor Value in Data Distance (TDNN): Let* $t_{tdnn}\left(s_{i},u_{j}\right)$ *and* $h_{tdnn}\left(s_{i},u_{j}\right)$ *be the nearest spatial neighbor values of node* $s_{i}$ *for the temperature value and the humidity value at time point* $u_{j}$ *in data distance, respectively.*

Similar to the SDNN, the data distance is applied in TDNN to exploit the relationship between different types of values acquired by nodes, but the difference is that the values used to calculate TDNN are obtained from the same node at different time points. Compared with TTNN, the final value calculated in TDNN is based on the values of its temporal nearest neighbors, i.e., the values at all these time points, rather than the values at two nearest time points used in TTNN. The data distance between two time points in the same window is denoted by $\;D_{td\;}\left(\cdot ,\cdot \right)$, to the temperature value; the data distance of nodes $s_{i}$ between two time points $u_{p}$ and $u_{q}$ can be computed as $\;D_{td\;}\left(u_{p},u_{q}\right)=\sqrt{{\left(h\left(s_{i},u_{p}\right)-h\left(s_{i},u_{q}\right)\right)}^{2}}=\left|h\left(s_{i},u_{p}\right)-h\left(s_{i},u_{q}\right)\right|$; likewise, to the humidity value, the data distance between $u_{p}$ and $u_{q}$ can be computed as $\;D_{td\;}\left(u_{p},u_{q}\right)=\sqrt{{\left(t\left(s_{i},u_{p}\right)-t\left(s_{i},u_{q}\right)\right)}^{2}}=\left|t\left(s_{i},u_{p}\right)-t\left(s_{i},u_{q}\right)\right|$.

Then the temporal nearest neighbor value of node $s_{i}$ for the temperature value at time point $u_{j}$ in data distance can be computed as

(21) $$t_{tdnn}\left(s_{i},u_{j}\right)\;=\left\{\begin{array}{cc} \underset{I\left(i,\;j\right)}{\sum }W_{td}\left(u_{j},u_{q}\right)t\left(s_{i},u_{q}\right) & \;\;h\left(s_{i},u_{j}\right)\;\neq NA\;and\;\forall \;u_{q}\in I\left(i,\;j\right),\;\exists h\left(s_{i},\;u_{q}\right)\;\neq NA\\ NA & \;\;h\left(s_{i},u_{j}\right)\;=NA\\ NA & \;\;\forall u_{q}\in I\left(i,\;j\right),\;h\left(s_{i},\;u_{q}\right)\;=NA \end{array}\right.$$

Likewise, the temporal nearest neighbor value of node $s_{i}$ for the humidity value at time point $u_{j}$ in data distance can be computed as

(22) $$h_{tdnn}\left(s_{i},u_{j}\right)\;=\left\{\begin{array}{cc} \underset{I\left(i,\;j\right)}{\sum }W_{td}\left(u_{j},u_{q}\right)h\left(s_{i},u_{q}\right) & \;\;t\left(s_{i},u_{j}\right)\;\neq NA\;and\;\forall \;u_{q}\in I\left(i,\;j\right),\;\exists t\left(s_{i},\;u_{q}\right)\;\neq NA\\ NA & \;\;t\left(s_{i},u_{j}\right)\;=NA\\ NA & \;\;\forall u_{q}\in I\left(i,j\right),t\left(s_{i},u_{q}\right)\;=NA \end{array}\right.$$

where $u_{q}\in I\left(i,j\right)$, and the weight $W_{td}$ can be computed as

(23) $$W_{td}\left(u_{j},u_{q}\right)\;=\frac{K\left(\;D_{td\;}\left(u_{j},u_{q}\right)\right)}{\sum_{I\left(i,\;j\right)}K\left(\;D_{td\;}\left(u_{j},u_{q}\right)\right)}$$

where $K\left(\cdot \right)$ is a kernel function, for example, Gaussian. 

Similar as the spatial nearest neighbor values in data distance, if $h\left(s_{i},u_{j}\right)$ or $t\left(s_{i},u_{j}\right)$ is missing, $t_{tdnn}\left(s_{i},u_{j}\right)$ or $h_{tdnn}\left(s_{i},u_{j}\right)\;$ will be unavailable, denoted by NA.

#### 3.2.2. Correlations between a Node’s Raw Value and Its Spatial (Temporal) Nearest Neighbor Values

**Figure 1:**
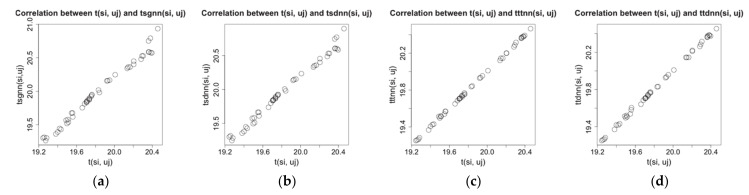
Correlation between raw temperature values and spatial (temporal) nearest neighbor values on Intel Lab dataset. (**a**) $\;t\left(s_{i},u_{j}\right)$ and $t_{sgnn}\left(s_{i},u_{j}\right)$. (**b**) $\;t\left(s_{i},u_{j}\right)$ and $t_{sdnn}\left(s_{i},u_{j}\right)$. (**c**) $t\left(s_{i},u_{j}\right)$ and $t_{ttnn}\left(s_{i},u_{j}\right)$. (**d**) $t\left(s_{i},u_{j}\right)$ and $t_{tdnn}\left(s_{i},u_{j}\right)$.

**Figure 2:**
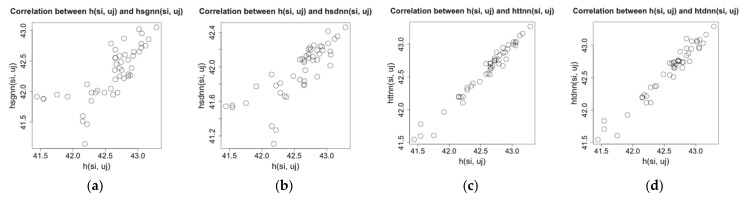
Correlation between raw humidity values and spatial (temporal) nearest neighbor values on Intel Lab dataset. (**a**) $\;h\left(s_{i},u_{j}\right)$ and $h_{sgnn}\left(s_{i},u_{j}\right)$. (**b**) $\;h\left(s_{i},u_{j}\right)$ and $h_{sdnn}\left(s_{i},u_{j}\right)$. (**c**) $h\left(s_{i},u_{j}\right)$ and $h_{ttnn}\left(s_{i},u_{j}\right)$. (**d**) $h\left(s_{i},u_{j}\right)$ and $h_{tdnn}\left(s_{i},u_{j}\right)$.

The node $s_{i}$ gets the temperature value $t\left(s_{i},u_{j}\right)$ and the humidity value $h\left(s_{i},u_{j}\right)$ at the time point $u_{j}$ on its location in the physical space. Here $t\left(s_{i},u_{j}\right)$ and $h\left(s_{i},u_{j}\right)$ are raw values. By data analysis, we find that the raw values of a node have strong relationship with their spatial (temporal) nearest neighbor values. For example, on the experimental dataset of Intel Lab, after choosing a time window W and a node $s_{i}$ randomly, we calculate spatial (temporal) nearest neighbor values for the node and compare them with the raw values $t\left(s_{i},u_{j}\right)$ and $h\left(s_{i},u_{j}\right)$; the results are shown as Figure 1 and Figure 2.

The Figure 1 and Figure 2 show that there are strong linear correlations between the raw values of a sensor node and their spatial (temporal) nearest neighbor values. Among them, the correlation between the raw values and temporal nearest neighbor values is higher than that between the raw values and spatial nearest neighbor values, but when we increase the sampling interval for the test data, the data density of the test dataset is decreased, and the correlation between the raw values and temporal nearest neighbor values becomes lower. In addition, the correlations on the humidity data are lower than those on the temperature data because the humidity has been affected by more complex environmental factors than the temperature. On another experimental dataset, the GreenOrbs [18], we can get the similar results on the correlations between raw values and spatial (temporal) nearest neighbor values. That means these correlations and their patterns of change, together with linear regression tool, can be applied to make estimation for missing values for a node. It is a new way to design the imputation algorithm. 

### 3.3. TSNN Imputation Algorithm

#### 3.3.1. The Methods to Calculate the Imputation Value

Here we consider the temperature value $t\left(s_{i},u_{j}\right)\;$ measured by node $s_{i}$ at time point $u_{j}$; it has four different nearest neighbor values, $t_{sgnn}\left(s_{i},u_{j}\right)$, $t_{sdnn}\left(s_{i},u_{j}\right)$, $t_{ttnn}\left(s_{i},u_{j}\right)$ and $t_{tdnn}\left(s_{i},u_{j}\right)$; the first two values come from spatial nearest neighbors in geometrical distance and in data distance, respectively, and the last two values come from the temporal nearest neighbors in time distance and in data distance, respectively.

Then the $t\left(s_{i},u_{j}\right)\;$ can be described in four different equations as

(24) $$\left\{\begin{array}{c} t\left(s_{i},u_{j}\right)\;=\alpha_{s_{i}}\left(1\right)+\beta_{s_{i}}\left(1\right)t_{sgnn}\left(s_{i},u_{j}\right)+noise\left(1\right)\\ t\left(s_{i},u_{j}\right)\;=\alpha_{s_{i}}\left(2\right)+\beta_{s_{i}}\left(2\right)t_{sdnn}\left(s_{i},u_{j}\right)+noise\left(2\right)\\ t\left(s_{i},u_{j}\right)\;=\alpha_{s_{i}}\left(3\right)+\beta_{s_{i}}\left(3\right)t_{ttnn}\left(s_{i},u_{j}\right)+noise\left(3\right)\\ t\left(s_{i},u_{j}\right)\;=\alpha_{s_{i}}\left(4\right)+\beta_{s_{i}}\left(4\right)t_{tdnn}\left(s_{i},u_{j}\right)+noise\left(4\right) \end{array}\right.$$

As in the discussion in Section 3.2.2, we can use linear regression to make estimation for missing values of a node. Supposed a temperature value measured by node $s_{i}$ at time point $u_{miss}$ is missing, $u_{miss}\in U_{miss}$, $U_{miss}\subset W$. We can find four estimated values for it, denoted by $\hat{t\left(s_{i},u_{miss}\right)\;}\left(1\right)$, $\hat{t\left(s_{i},u_{miss}\right)\;}\left(2\right)$, $\hat{t\left(s_{i},u_{miss}\right)\;}\left(3\right)$ and $\hat{t\left(s_{i},u_{miss}\right)\;}\left(4\right)$. They can be computed as

(25) $$\left\{\begin{array}{c} \hat{t\left(s_{i},u_{miss}\right)}\;\left(1\right)\;=\alpha_{s_{i}}\left(1\right)+\beta_{s_{i}}\left(1\right)t_{sgnn}\left(s_{i},u_{miss}\right)\\ \hat{t\left(s_{i},u_{miss}\right)}\;\left(2\right)\;=\alpha_{s_{i}}\left(2\right)+\beta_{s_{i}}\left(2\right)t_{sdnn}\left(s_{i},u_{miss}\right)\\ \hat{t\left(s_{i},u_{miss}\right)}\;\left(3\right)\;=\alpha_{s_{i}}\left(3\right)+\beta_{s_{i}}\left(3\right)t_{ttnn}\left(s_{i},u_{miss}\right)\\ \hat{t\left(s_{i},u_{miss}\right)}\;\left(4\right)\;=\alpha_{s_{i}}\left(4\right)+\beta_{s_{i}}\left(4\right)t_{tdnn}\left(s_{i},u_{miss}\right) \end{array}\right.$$

Applying the spatial nearest neighbor value of node $s_{i}$ at time point $u_{miss}$ in geometrical distance, we have the estimated value, denoted by $\hat{t\left(s_{i},u_{miss}\right)\;}\left(1\right)$; the model is

(26) $$\hat{t\left(s_{i},u_{miss}\right)}\;\left(1\right)\;=\alpha_{s_{i}}\left(1\right)+\beta_{s_{i}}\left(1\right)t_{sgnn}\left(s_{i},u_{miss}\right)$$

$\alpha_{s_{i}}\left(1\right)$ and $\beta_{s_{i}}\left(1\right)$ can be computed by minimized residual sum of squares as

(27) $$\alpha_{s_{i}}\left(1\right),\beta_{s_{i}}\left(1\right)\;=\underset{\alpha_{s_{i}}{}^{’}\left(1\right),\beta_{s_{i}}{}^{’}\left(1\right)}{\mathrm{argmin}}\underset{U_{sample}}{\sum }{\left(t\left(s_{i},u_{sample}\right)\left(1\right)-\alpha_{s_{i}}{}^{’}\left(1\right)-\beta_{s_{i}}{}^{’}\left(1\right)t_{sgnn}\left(s_{i},u_{sample}\right)\right)}^{2}$$

where $u_{sample}$ is the sample time points from non-missing data in the window W, $U_{sample}\subset W$, $U_{sample}\cap U_{miss}=\phi$, $u_{sample}\in U_{sample}$.

Meanwhile, the linear relationship between $t\left(s_{i},u_{sample}\right)\left(1\right)$ and $\hat{t\left(s_{i},u_{sample}\right)\left(1\right)}$ can be measured by $R^{2}$ as

(28) $$R_{si}^{2}\left(1\right)\;=\frac{\sum_{U_{sample}}{\left(\hat{t\left(s_{i},u_{sample}\right)\left(1\right)\;}-\bar{t\left(s_{i},u_{sample}\right)\left(1\right)}\;\right)}^{2}}{\sum_{U_{sample}}{\left(t\left(s_{i},u_{sample}\right)\left(1\right)\;-\bar{t\left(s_{i},u_{sample}\right)\left(1\right)}\;\right)}^{2}}$$

Similarly, applying the spatial nearest neighbor value of node $s_{i}$ at time point $u_{miss}$ in data distance, we have another estimated value, denoted by $\hat{t\left(s_{i},u_{miss}\right)\;}\left(2\right)$*,* the model is

(29) $$\hat{t\left(s_{i},u_{miss}\right)}\left(2\right)\;=\alpha_{s_{i}}\left(2\right)+\beta_{s_{i}}\left(2\right)t_{sdnn}\left(s_{i},u_{miss}\right)$$

where

(30) $$\alpha_{s_{i}}\left(2\right),\beta_{s_{i}}\left(2\right)\;=\underset{\alpha_{s_{i}}{}^{’}\left(2\right),\beta_{s_{i}}{}^{’}\left(2\right)}{\mathrm{argmin}}\underset{U_{sample}}{\sum }{\left(t\left(s_{i},u_{sample}\right)\left(1\right)-\alpha_{s_{i}}{}^{’}\left(2\right)-\beta_{s_{i}}{}^{’}\left(2\right)t_{sdnn}\left(s_{i},u_{sample}\right)\right)}^{2}$$

where $U_{sample}\subset W$, $U_{sample}\cap U_{miss}=\phi$, $u_{sample}\in U_{sample}$.

The linear relationship between $t_{s_{i},u_{sample}}\left(2\right)$ and $\hat{t_{s_{i},u_{sample}}}\left(2\right)$ can be measured by $R^{2}$ as

(31) $$R_{si}^{2}\left(2\right)\;=\frac{\sum_{U_{sample}}{\left(\hat{t\left(s_{i},u_{sample}\right)\left(2\right)\;}-\bar{t\left(s_{i},u_{sample}\right)\left(2\right)}\;\right)}^{2}}{\sum_{U_{sample}}{\left(t\left(s_{i},u_{sample}\right)\left(2\right)\;-\bar{t\left(s_{i},u_{sample}\right)\left(2\right)}\;\right)}^{2}}$$

The estimated value using spatial nearest neighbor values of node $s_{i}$ at time point $u_{miss}$, denoted by $\hat{t\left(s_{i},u_{miss}\right)\;}\left(S\right)$, can be computed as

(32) $$\hat{t\left(s_{i},u_{miss}\right)}\left(S\right)\;=\left\{\begin{array}{cc} \frac{R_{si}^{2}\left(1\right)}{R_{si}^{2}\left(1\right)+R_{si}^{2}\left(2\right)}\hat{t\left(s_{i},u_{miss}\right)}\left(1\right)+\frac{R_{si}^{2}\left(2\right)}{R_{si}^{2}\left(1\right)+R_{si}^{2}\left(2\right)}\hat{t\left(s_{i},u_{miss}\right)}\left(2\right) & t_{sdnn}\left(s_{i},u_{miss}\right)\;\neq \;NA,t_{sdnn}\left(s_{i},u_{miss}\right)\;\neq \;NA\\ \hat{t\left(s_{i},u_{miss}\right)}\left(S\right)\;=\;\hat{t\left(s_{i},u_{miss}\right)}\left(1\right) & t_{sdnn}\left(s_{i},u_{miss}\right)\;=\;NA\\ NA & t_{sdnn}\left(s_{i},u_{miss}\right)\;=\;NA \end{array}\right.$$

Following the similar steps, the estimated value using temporal nearest neighbor values of node $s_{i}$ at time point $u_{miss}$, denoted by $\hat{t\left(s_{i},u_{miss}\right)\;}\left(T\right)$, can be computed as

(33) $$\hat{t\left(s_{i},u_{miss}\right)}\left(T\right)\;=\left\{\begin{array}{cc} \frac{R_{si}^{2}\left(3\right)}{R_{si}^{2}\left(3\right)+R_{si}^{2}\left(4\right)}\hat{t\left(s_{i},u_{miss}\right)}\left(3\right)+\frac{R_{si}^{2}\left(4\right)}{R_{si}^{2}\left(3\right)+R_{si}^{2}\left(4\right)}\hat{t\left(s_{i},u_{miss}\right)}\left(4\right) & t_{sdnn}\left(s_{i},u_{miss}\right)\;\neq \;NA,t_{sdnn}\left(s_{i},u_{miss}\right)\;\neq \;NA\\ \hat{t\left(s_{i},u_{miss}\right)}\left(S\right)\;=\;\hat{t\left(s_{i},u_{miss}\right)}\left(3\right) & t_{sdnn}\left(s_{i},u_{miss}\right)\;=\;NA\\ NA & t_{sdnn}\left(s_{i},u_{miss}\right)\;=\;NA \end{array}\right.$$

The final estimated temperature value for node $s_{i}$ at time point $u_{miss}$ is denoted by $\hat{t\left(s_{i},u_{miss}\right)}$, which can be computed based on the two values: $\hat{t\left(s_{i},u_{miss}\right)\;}\left(S\right)$ and $\hat{t\left(s_{i},u_{miss}\right)\;}\left(T\right)$. The contributions of the two values are different to calculate the final estimation for the node $s_{i}$ at time point $u_{miss}$, which are determined by the physical environment factors in the time window W of the space P, where the node $s_{i}\;$ owns its spatial and temporal nearest neighbors. In fact, the experiment results demonstrate that the contribution ratio basically keeps unchanged in the observed time window. Therefore, a spatial–temporal coefficient, denoted by λ, can be used to describe the contribution ratio of the $\hat{t\left(s_{i},u_{miss}\right)\;}\left(S\right)$ and $\hat{t\left(s_{i},u_{miss}\right)\;}\left(T\right)$, which will be discussed in the next section.

The final estimated temperature value for node $s_{i}$ at time point $u_{miss}$, denoted by $\hat{t\left(s_{i},u_{miss}\right)\;}$ can be computed as

(34) $$\hat{t\left(s_{i},u_{miss}\right)}=\left\{\begin{array}{cc} \hat{\lambda t\left(s_{i},u_{miss}\right)}\left(S\right)+(1-\lambda )t\hat{\left(s_{i},u_{miss}\right)}\left(T\right) & \hat{t\left(s_{i},u_{miss}\right)}\left(S\right)\;\neq \;NA,\hat{t\left(s_{i},u_{miss}\right)}\left(T\right)\;\neq \;NA\\ \hat{t\left(s_{i},u_{miss}\right)}\left(S\right) & \hat{t\left(s_{i},u_{miss}\right)}\left(S\right)\;\neq \;NA,\hat{t\left(s_{i},u_{miss}\right)}\left(T\right)\;=\;NA\\ \hat{t\left(s_{i},u_{miss}\right)}\left(T\right) & \hat{t\left(s_{i},u_{miss}\right)}\left(S\right)\;\neq \;NA,\hat{t\left(s_{i},u_{miss}\right)}\left(T\right)\;\neq \;NA\\ NA & \hat{t\left(s_{i},u_{miss}\right)}\left(S\right)\;=\;NA \end{array}\right.$$

Then, $\hat{t\left(s_{i},u_{miss}\right)\;}\;$ will be used as the estimated value for $\;t\left(s_{i},u_{miss}\right)$.

The implementation of TSNN is shown in Algorithm 1.

**Algorithm 1:** TSNN algorithm. **Input:** sensor node $s_{i},\;s_{i}\in S;\;u_{miss},\;u_{miss}\in W$; temperature dataset $;\;\;\;T\left(S,W\right)$, humidity dataset $H\left(S,W\right),$    $S=\left\{s_{1},s_{2},\ldots ,s_{n-1},s_{n}\right\},\;W=\left\{u_{1},u_{2},\ldots ,u_{m-1},u_{m}\right\}$ initial number of spatial nearest neighbors $k_{s};$    initial number of temporal nearest neighbors $k_{t};$ the spatial—temporal coefficient λ. **Output:**
$\hat{t\left(s_{i},u_{miss}\right).}$1. Get $k_{sb}$ and $k_{tb}$2. $k_{s}\leftarrow k_{sb},\;k_{t}\leftarrow k_{tb}$3. $T^{\prime }\left(S,W^{\prime }\right),H^{\prime }\left(S,W^{\prime }\right)\leftarrow all\;elements\;in\;T\left(S,W\right),H\left(S,W\right)$ which do not contain missing values for node $s_{i}$4. $T^{\prime \prime }\left(S,W^{\prime \prime }\right),H^{\prime \prime }\left(S,W^{\prime \prime }\right)\leftarrow time\;sampling\;\;from\;\;T^{\prime }\left(S,W^{\prime }\right),H^{\prime }\left(S,W^{\prime }\right)$5. $m^{\prime \prime }\leftarrow \left|W^{\prime \prime }\right|$6. A←∅, B←∅, C←∅, D←∅, E←∅7. **for**
*j* from 1 to $m^{\prime \prime }$ do8.   On $\;\mathrm{On}\;T^{\prime \prime }\left(S,W^{\prime \prime }\right)\;and\;H^{\prime \prime }\left(S,W^{\prime \prime }\right),$ get $t_{sgnn}\left(s_{i},u_{j}\right),\;t_{sdnn}\left(s_{i},u_{j}\right),\;t_{ttnn}\left(s_{i},u_{j}\right)\;\mathrm{and}\;t_{tdnn}\left(s_{i},u_{j}\right)$9.  $A\leftarrow A+\left\{t\left(s_{i},u_{j}\right)\right\}$10.  **if**
$t_{sgnn}\left(s_{i},u_{j}\right)\neq NA\;$**then**11.    $B\leftarrow B+\left\{t_{sgnn}\left(s_{i},u_{j}\right)\right\}$12.  **else if**
$t_{sdnn}\left(s_{i},u_{j}\right)\neq NA$
**then**13.    $C\leftarrow C+\left\{t_{sdnn}\left(s_{i},u_{j}\right)\right\}$14.  **else if**
$t_{ttnn}\left(s_{i},u_{j}\right)\neq NA$
**then**15.    $D\leftarrow D+\left\{t_{ttnn}\left(s_{i},u_{j}\right)\right\}$16.   **else if**
$t_{tdnn}\left(s_{i},u_{j}\right)\neq NA$
**then**17.    $E\leftarrow E+\left\{t_{tdnn}\left(s_{i},u_{j}\right)\right\}$18.  **end if**19. **end for**20. On $T\left(S,W\right)\;and\;H\left(S,W\right),\;$ get $t_{sgnn}\left(s_{i},u_{miss}\right),\;t_{sdnn}\left(s_{i},u_{miss}\right),\;t_{ttnn}\left(s_{i},u_{miss}\right)$ and $t_{tdnn}\left(s_{i},u_{miss}\right)$21. **if**
$t_{sgnn}\left(s_{i},u_{miss}\right)=NA$ and $t_{ttnn}\left(s_{i},u_{miss}\right)=NA$
**then**22.  $\hat{t\left(s_{i},u_{miss}\right)\;}\leftarrow NA$23.  **return**
$\hat{t\left(s_{i},u_{miss}\right)\;}$24. **else if**
$t_{sgnn}\left(s_{i},u_{miss}\right)\neq NA$
**then**25.  Construct the estimation equation $E\left(1\right)\left(s_{i},u_{miss}\right)$26.  Using *A* and *B*, regress the coefficients $\alpha_{s_{i}}\left(1\right),\beta_{s_{i}}\left(1\right)$ of $E\left(1\right)\left(s_{i},u_{miss}\right)$ and compute $R_{si}^{2}\left(1\right)$27. **else if**
$t_{sdnn}\left(s_{i},u_{miss}\right)\neq NA$
**then**28.  Construct the estimation equation $E\left(2\right)\left(s_{i},u_{miss}\right)$29.  Using *A* and *C*, regress the coefficients $\alpha_{s_{i}}\left(2\right),\beta_{s_{i}}\left(2\right)$ of $E\left(2\right)\left(s_{i},u_{miss}\right)$ and compute $R_{si}^{2}\left(2\right)$30. **else if**
$t_{ttnn}\left(s_{i},u_{miss}\right)\neq NA$
**then**31.  Construct the estimation equation $E\left(3\right)\left(s_{i},u_{miss}\right)$32.  Using *A and D*, regress the coefficients $\alpha_{s_{i}}\left(3\right),\beta_{s_{i}}\left(3\right)$ of $E\left(3\right)\left(s_{i},u_{miss}\right)$ and compute $R_{si}^{2}\left(3\right)$33. **else if**
$t_{tdnn}\left(s_{i},u_{miss}\right)\neq NA$
**then**34.  Construct the estimation equation $E\left(4\right)\left(s_{i},u_{miss}\right)$35.  Using *A* and *E*, regress the coefficients $\alpha_{s_{i}}\left(4\right),\beta_{s_{i}}\left(4\right)$ of $E\left(4\right)\left(s_{i},u_{miss}\right)$ and compute $R_{si}^{2}\left(4\right)$36. **end if**37. Compute the $\hat{t\left(s_{i},u_{miss}\right)\;}\left(1\right),\;\hat{t\left(s_{i},u_{miss}\right)}\left(2\right)\;\;to\;\mathrm{get}\;\hat{t\left(s_{i},u_{miss}\right)\;}\left(S\right)$38. Compute the $\hat{t\left(s_{i},u_{miss}\right)\;}\left(3\right),\;\hat{t\left(s_{i},u_{miss}\right)}\left(4\right)\;\;\mathrm{to}\;\mathrm{get}\;\hat{t\left(s_{i},u_{miss}\right)\;}\left(T\right)$39. Compute the $\hat{t\left(s_{i},u_{miss}\right)\;}$ using $\hat{t\left(s_{i},u_{miss}\right)\;}\left(S\right),\;\hat{t\left(s_{i},u_{miss}\right)\;}\left(T\right)$ and λ40. **return**
$\hat{t\left(s_{i},u_{miss}\right)\;}$

#### 3.3.2. The Spatial–Temporal Coefficient λ

In the observed time window W, the value measured by node $s_{i}$ at time point $u_{miss}$ is missing, $u_{miss}\in U_{miss}$, $U_{miss}\subset W$. The experiment results demonstrate that the contribution ratio of the $\hat{t\left(s_{i},u_{miss}\right)\;}\left(S\right)$ and $\hat{t\left(s_{i},u_{miss}\right)\;}\left(T\right)\;$ basically keeps unchanged in the time window. Therefore, the spatial–temporal coefficient λ in time window W can be obtained based on the non-missing test data in the window. We put non-missing test data into different new assistant window $V_{i}$, all assistant windows form a windows group, denoted by  VG, $VG=\left\{V_{1},V_{2},\ldots ,V_{r-1},V_{r}\right\}\;$, $V_{i}\in VG$, $V_{i}=\left\{v_{1},v_{2},\ldots ,v_{m-1},v_{m}\right\}$,$\;V_{i}\subset W$, $V_{i}\cap U_{miss}=\emptyset$. Then we can generate a dataset with hypothetical random missing values based on time window $V_{i}$. For each raw value $t\left(s_{i},v_{miss}\right)\;\;$ marked as a missing value in the dataset, we will calculate its estimated values as the $\hat{t\left(s_{i},v_{miss}\right)\;}\left(S\right)$ and $\hat{t\left(s_{i},v_{miss}\right)\;}\left(T\right)\;$ using Equations (32) and (33). In the next step, for all missing values in time window $V_{i}$, using the equation 40, we can calculate the spatial and temporal RMSE values for the given window $V_{i}$, which is denoted by $RMSE\left(S,V_{i}\right)$ and $RMSE\left(T,V_{i}\right),$ respectively.

Then the spatial–temporal coefficient λ can be computed as

(35) $$\lambda =\frac{\sum_{VG}N\left(S,V_{i}\right)}{\sum_{VG}\left(N\left(S,V_{i}\right)+N\left(T,V_{i}\right)\right)}$$

where

(36) $$N\left(S,V_{i}\right)\;=\left\{\begin{array}{cc} \;1 & if\;RMSE\left(S,V_{i}\right)\leq RMSE\left(T,V_{i}\right)\\ \;0 & if\;RMSE\left(S,V_{i}\right)>RMSE\left(T,V_{i}\right) \end{array}\right.$$

(37) $$N\left(T,V_{i}\right)\;=\left\{\begin{array}{cc} \;1 & if\;RMSE\left(T,V_{i}\right)\leq RMSE\left(S,V_{i}\right)\\ \;0 & if\;RMSE\left(T,V_{i}\right)>RMSE\left(S,V_{i}\right) \end{array}\right.$$

where $V_{i}\in VG,\;VG=\left\{V_{1},V_{2},\ldots ,V_{r-1},V_{r}\right\}$.

#### 3.3.3. The Best $k_{s}$ for Spatial Nearest Neighbors and $k_{t}$ for Temporal Nearest Neighbors

As in discussion in Section 3.3.1, before computing the final estimated value the $\hat{t\left(s_{i},u_{miss}\right)\;}$, we must get the spatial nearest neighbor value in data distance $t_{sdnn}\left(s_{i},u_{miss}\right)$ and temporal nearest neighbor value in data distance $t_{tdnn}\left(s_{i},u_{miss}\right)$. The two values can be worked out based on the values of their neighbors at the time point, respectively, that is, $k_{s}$ spatial nearest neighbors in $G\left(i,miss\right)$ and $k_{t}$ temporal nearest neighbors in $I\left(i,miss\right)$. The experimental results show that different $k_{s}$ and $k_{t}$ will have different effects to the deviation between $t_{sdnn}\left(s_{i},u_{j}\right)$, $t_{tdnn}\left(s_{i},u_{j}\right)$ and the real value $t\left(s_{i},u_{j}\right)$; meanwhile, there exist the best $k_{s}$ and $k_{t}$ which make the least deviations. Basically, in an observed time window with missing values at some time points, the relationships between the real value $t\left(s_{i},u_{j}\right)$ and its spatial nearest neighbor value $t_{sdnn}\left(s_{i},u_{j}\right)$ or its temporal nearest neighbor value $t_{tdnn}\left(s_{i},u_{j}\right)$ keep stable; therefore, the best $k_{s}$ and $k_{t}$ obtained by the samples from non-missing values will still make the least deviation when they are applied to calculate neighbor values $t_{sdnn}\left(s_{i},u_{miss}\right)\;$ and $t_{tdnn}\left(s_{i},u_{miss}\right)$ for the missing value. 

In the observed time window W, let $T\left(s_{i},U_{sample}\right)$ be the vector composed of $\;t\left(s_{i},u_{sample}\right)$, $T_{sdnn}\left(s_{i},k_{s}^{’},U_{sample}\right)\;$ be the vector composed of $t_{sdnn}\left(s_{i},k_{s}^{’},u_{sample}\right)\;$ which is computed based on $k_{s}^{’}$ and $T_{tdnn}\left(s_{i},k_{t}^{’},U_{sample}\right)$ be the vector composed of $t_{tdnn}\left(s_{i},k_{t}^{’},u_{sample}\right),$ which is computed based on $k_{t}^{’}$. Given the initial $k_{s}$ and $k_{t}$, the best $k_{s}$ and $k_{t}$ can be computed as

(38) $$k_{sb}=\underset{k_{s}^{’}}{\mathrm{argmax}}Pearson\left(T_{sdnn}\left(s_{i},k_{s}^{’},U_{sample}\right),T\left(s_{i},U_{sample}\right)\right)$$

(39) $$k_{tb}=\underset{k_{t}^{’}}{\mathrm{argmax}}Pearson\left(T_{tdnn}\left(s_{i},k_{t}^{’},U_{sample}\right),T\left(s_{i},U_{sample}\right)\right)$$

where $Pearson\left(\cdot \right)$ is the function used to calculate the Pearson relationship coefficient between two vectors.

## 4. Experimental Results

### 4.1. Evaluation Platform Used in Experiments

To make the evaluation for our algorithm, all experiments are running on a laptop with Intel Core i7 2.9 GHz CPU and 16 GB RAM. The codes are implemented by R language. 

### 4.2. Evaluation Methods Used in Experiments

Two measuring sticks have been applied for evaluating the experimental results. The first one is the root mean square error (RMSE), which will be used to measure the accuracy of the estimation for missing values. The other one is the percentage of cases in which a mission value can be estimated (PCE), which will be used to measure the applicability of the imputation algorithm in a real application.

The RMSE is computed as

(40) $$RMSE=\;\sqrt{\frac{\sum_{}{\left(real\;value\;-\;estimated\;value\right)}^{2}}{the\;numer\;of\;estimations}}$$

The PCE is computed as:

(41) $$PCE\left(\%\right)\;=\;\frac{the\;number\;of\;cases\;that\;a\;missing\;value\;can\;be\;estimated}{the\;total\;number\;of\;attempts\;to\;estimate\;a\;missing\;value}*100\%$$

### 4.3. Experimental Datasets

We test our TSNN algorithm and other algorithms based on two real world datasets: the Intel Lab dataset and the GreenOrbs dataset. They represent two kinds of typical real wireless network: the indoor network with unchanged common spatial neighbors and the outdoor network with changing common spatial neighbors. The nodes deployed in the two different WSNs have similar structures that each node is equipped with more than one sensor for obtaining different physical quantities at their locations. In addition, all the nodes are working continuously in the real environment. The datasets provide an ideal platform for experiments where our algorithm can obtain and exploit the information hidden in the data. Moreover, the two WSNs have different topological structures for the node to build its neighbors, which make the node in the different datasets have fixed neighbors and unfixed neighbors, respectively, and this change can be adapted by our algorithm and does not degrade its performance.

#### 4.3.1. Intel Lab Dataset

Intel Lab dataset contains indoor data collected from 54 sensor nodes deployed in the Intel Berkeley Research lab. Each sensor node will get the data including temperature, humidity, light and voltage values once every 31 s. To the data we collected using the in-network query processing system, a monotonically increasing sequence number is applied to make sure the data processing center can get the data from different nodes at the same time point. We only use the temperature and humidity data in our experiments. In the raw dataset, data at some time points on some nodes have not been recorded. When we evaluate applicability of imputation algorithms using PCE and the accuracy of imputation algorithms using RMSE on the real-world dataset, all data in the raw dataset are used directly; in other words, the raw dataset is used as the experimental dataset. 

In this paper, we consider the situation that the test node has missing values at time points randomly selected in the time window. Therefore, in the following experiments, some data of the test node have been marked as dummy missing values (not available, denoted by NA) following the Missing Completely at Random (MCAR) [19]. In real situations, the data coming from the temperature sensor and the humidity sensor on the same node will have three possible scenarios: the temperature (humidity) data are missing but the humidity (temperature) data are available at all time points; the temperature (humidity) data are missing but the humidity (temperature) data are available at some of the points in certain probability, and the temperature and humidity data are both missing at all time points. Accordingly, in the experiments, the three test scenarios have been defined as follows: For the test noted at the time point, humidity (temperature) value is not NA when temperature (humidity) value is marked as NA; humidity (temperature) value is NA in certain probability (set as 50% in the experiments) when temperature (humidity) value is marked as NA; humidity value is NA when temperature value is marked as NA. Next, we will apply imputation algorithms to estimate missing temperature (humidity) values and compare them with real values.

**Figure 3:**
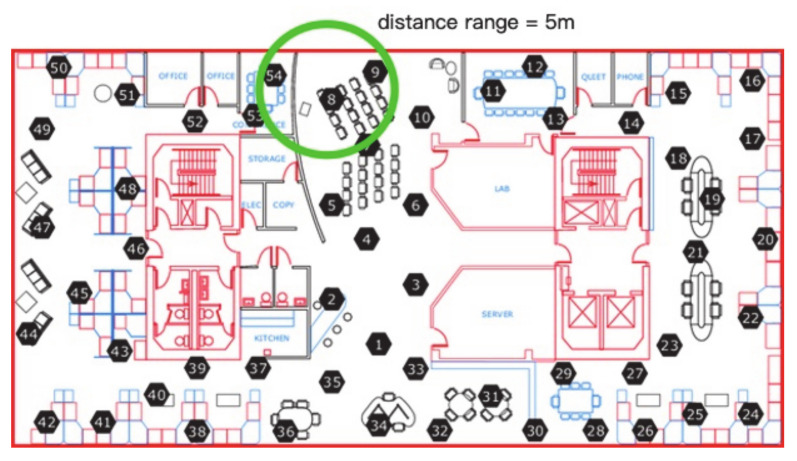
The example of a neighbor group affected by walls in Intel Lab dataset.

In the Intel Lab dataset, the common spatial neighbors will keep unchanged at time points in a time window. As one of the parameters used in algorithm, the initial number of neighbors in the group will be decided by distance range between the test node and its neighbor node as well as whether there is a wall or door between nodes when creating a neighbor group for the test node. For example, the distance between node 8 and node 54 is 2.83 m, and the distance between node 8 and node 9 is 3.61 m, when the distance range is set as 5 m, the node 9 and node 54 should both be the neighbor nodes of node 8, but the wall between the node 54 and node 8 will make node 54 excluded from the neighbor group of node 8, as Figure 3 shows. 

#### 4.3.2. GreenOrbs Dataset

**Figure 4:**
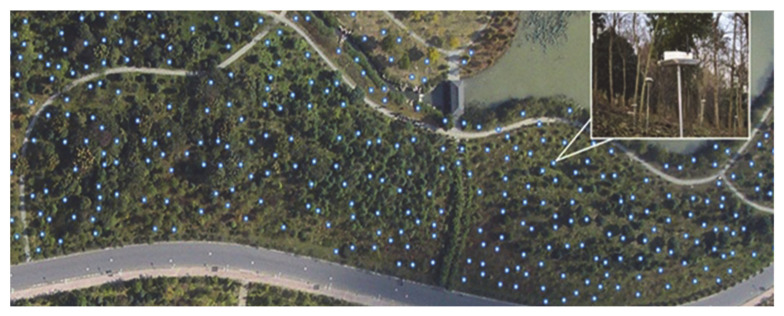
Wireless sensor nodes in GreenOrbs dataset.

GreenOrbs dataset contains outdoor data collected from 120 wireless sensor nodes scattered in the forest, shown in Figure 4. Each sensor node will get the data including temperature, humidity and light values once every 80 to 85 s. In experiments, we only use the temperature and humidity data. Similar as the Intel Lab dataset, the raw GreenOrbs dataset is not integrated; data at some time points from some nodes have not been recorded. Similarly, the raw dataset will be used as the experimental dataset directly when we evaluate applicability of imputation algorithms using PCE and the accuracy of imputation algorithms using RMSE.

Similar as experiment on Intel Lab dataset, some data of the test nodes have been marked as dummy missing values following MCAR in the three test scenarios before testing different imputation algorithms.

In the GreenOrbs dataset, different from the Intel Lab dataset, the neighbor group of a node will change in a time window because the neighbors are obtained according to the sensed RSSI values [20]. For example, the neighbors group of node 3 is {51, 7,2, 59, 87, 70, 50, 75, 4, 37, 95}, {72, 29, 70, 50, 120, 75, 113, 4, 37, 95} and {29, 70, 50, 75, 113, 4, 6, 84} at three continuous time points in the time window. Therefore the common spatial neighbors at these time points are {70, 50, 75, 4}. 

### 4.4. Evaluation of PCE

**Figure 5:**
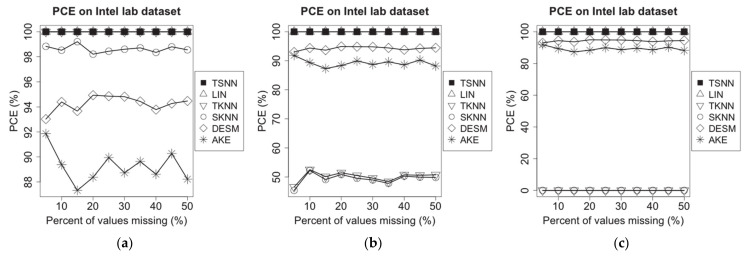
PCE of different algorithms on Intel lab dataset for the three scenarios: (**a**) temperature NA and humidity not NA, (**b**) temperature NA and humidity 50% NA and (**c**) temperature NA and humidity NA.

On the experimental dataset of Intel Lab, we choose a time window randomly, in which some of the temperature measuring data are supposed to be missing. Then, we randomly choose 20% of all nodes as test nodes; next, a missing percentage is applied to mark some of measuring data as lost status using MCAR method for one of the nodes. Because over 50% of data missing is risky for observed values, and imputation should not be used in this situation [21], the upper bound of missing percentage is set as 50% in our experiments for both PCE and RMSE evaluations. Then, on the three test scenarios, we apply different imputation algorithms to make the estimation for the missing values, each one as a case. The experiment has been repeated for each note, and the PCE can be calculated. Finally, for different imputation algorithms, we will work out the average PCE for all test nodes on each of missing percentages from 5% to 50%. The experimental results are shown in Figure 5.

**Figure 6:**
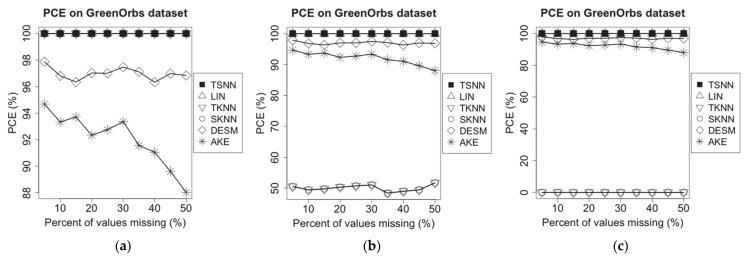
PCE of different algorithms on GreenOrbs dataset for the three scenarios: (**a**) temperature NA and humidity not NA, (**b**) temperature NA and humidity 50% NA and (**c**) temperature NA and humidity NA.

On GreenOrbs dataset, the same experiments have been made, and the results are shown in Figure 6.

Figure 5 shows that TSNN and LIN are able to make imputation for all cases on all the three scenarios because LIN only uses the temporal neighbor values, and TSNN only uses the temporal nearest neighbor value in time distance when other three values are unavailable. In other words, as long as there are at least two neighbor values in the time window, the preconditions of TSNN and LIN can be satisfied, and these two algorithms can work correctly. Actually, in our experiments, even if the percentage of values missing reaches the upper bound of 50%, the node still owns more than two temporal neighbor values in the time window; therefore, TSNN and LIN can make imputation in all cases and are not affected by the percentage of values missing. However, TKNN, SKNN, AKE and DESM cannot make imputation for some of the scenarios or some of the cases. TKNN is able to make imputation for all cases in scenario (a), but its PCE drops down to about 50% in scenario (b) and becomes unavailable in scenario (c) because the temporal nearest neighbor value in data distance on which it relied decreases in scenario (b) until unavailable in scenario (c). SKNN, AKE and DESM all need non-missing values coming from spatial neighbors of the test node in the test time window. However, on Intel lab dataset, for the whole test window, almost all nodes have some time points where the raw values are not recorded. In other words, although spatial neighbors existed throughout the entire time window, there are not raw values recorded on some of the time points. SKNN, DESM and AKE require at least one neighbor with non-missing values; besides, for the test note, DESM and AKE require at least one common spatial neighbor with non-missing values on at least two and three time points in the window; in scenarios (a), (b) and (c), both DESM and AKE have the same performance. In scenario (a), SKNN has better performance than DESM and AKE, but similar to TKNN; its PCE drops down over 50% in scenarios (b) and becomes unavailable in scenario (c) because the spatial nearest neighbor value in data distance on which it relied decreases in scenario (b) until unavailable in scenario (c).

Because the time points without recorded values are spread mainly evenly in the raw dataset, all algorithms have slightly changed as the percent of missing values increases.

On the experimental dataset of GreenOrbs, the situation is different from Intel lab dataset because the common spatial neighbors for a test node may be unavailable on a test window due to the neighbors being obtained according to the sensed RSSI values. It may cause one or more common spatial neighbors not to be available at time points selected randomly on the test window, which will make DESM or AKE not applicable in this case, and the degree of PCE degradation for AKE is the most prominent as the percent of missing values increases because of the increasing difficulty to get common spatial neighbors among less time points without missing values. However, in all three scenarios, SKNN always can get at least one spatial neighbor, so it has the same performance as TKNN, which always can get at least one temporal neighbor. Figure 6 shows TSNN and LIN are still able to make imputation for all cases on all three scenarios. 

Because the applicability of the imputation algorithms is only related to temporal and spatial neighbors, to humidity measurement data on Intel Lab dataset and GreenOrbs dataset, we can obtain the similar experimental results.

The experimental results on two test datasets show that TSNN has the best PCE performance.

### 4.5. Evaluation of RMSE

**Figure 7:**
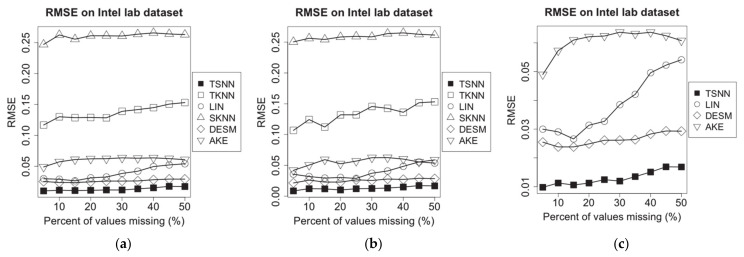
RMSE of imputation algorithms on temperature data of Intel lab dataset for the three scenarios: (**a**) temperature NA and humidity not NA, (**b**) temperature NA and humidity 50% NA and (**c**) temperature NA and humidity NA.

On the experimental dataset of Intel Lab dataset, the sampling interval is set to 1min, and we choose a time window randomly, on which some of the temperature measurement data are supposed to be missing. After that, we randomly choose 20% of all nodes as test nodes. Next, a missing percentage is applied to make the MCAR operation for one of the nodes; then, on the three test scenarios, we apply different imputation algorithms to make the estimation for the missing values, each one as a case. The experiment will be repeated for each test note. In experiments, we will compare the performance of TSNN with other imputation algorithms based on the common cases in which a missing value can be estimated by all algorithms, although they have different PCE. Finally, based on the test results of all cases, for different imputation algorithms, we work out the average RMSE for all test nodes on each of missing percentages from 5% to 50%. The experimental results have been shown in Figure 7.

For TSNN algorithm, the spatial–temporal coefficient λ, the best $k_{s}$ for spatial nearest neighbors and $k_{t}$ for temporal nearest neighbors will be calculated and applied to the algorithm. For TKNN algorithm, which requires $k_{t}$ for temporal nearest neighbors, and SKNN, DESM and AKE algorithms, which require $k_{s}$ for spatial nearest neighbors, they will use their own methods to select the suitable $k_{t}$ or $k_{s}$ to make the algorithm get the best performance in the experiment case.

**Figure 8:**
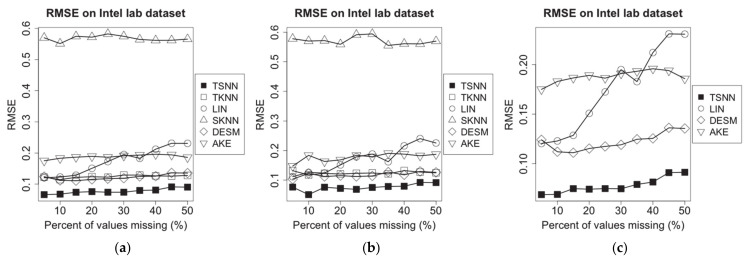
RMSE of imputation algorithms on humidity data of Intel lab dataset for the three scenarios: (**a**) humidity NA and temperature not NA, (**b**) humidity NA and temperature 50% NA and (**c**) humidity NA and temperature NA.

Supposing that the humidity measurement data are missing in the time window, we repeated the same experiments, and the results are shown in Figure 8.

**Figure 9:**
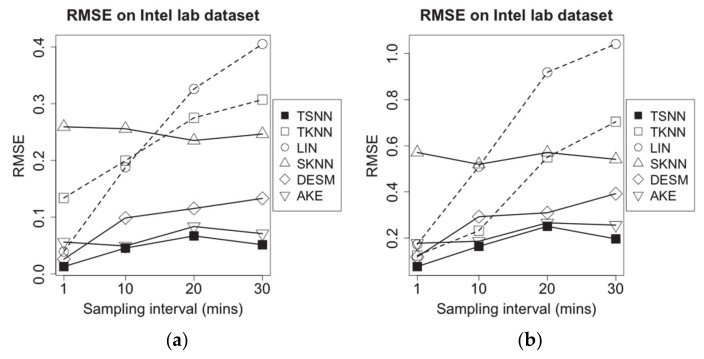
RMSE of imputation algorithms on different data density for Intel lab dataset: (**a**) temperature data and (**b**) humidity data.

To evaluate the performance of algorithms on different data density, we extract data from the experimental dataset based on different sampling intervals from 1 min to 30 min, to make the new test datasets and repeat the same experiments. To ensure that there is still enough data when we make sampling on the long interval, before making the new test datasets, the data that have not been recorded at some time points on some nodes in the raw dataset will be filled with the average of the non-missing value nearby. We test all algorithms on the most typical scenario: The temperature (humidity) data are missing, but the humidity (temperature) data are available at some of the time points in certain probability which is set as 50% in the experiments. For different imputation algorithms, we work out the average RMSE for all test nodes on all missing percentages from 5% to 50%. The experimental results have been shown in Figure 9.

Figure 7 shows experimental results for missing temperature measurement data. We find that TKNN and SKNN become unavailable in scenario (c). The RMSE for all imputation algorithms fluctuates as the percent of missing values increases; LIN, TKNN and SKNN do not use spatial or temporal correlation information; LIN has relatively lower RMSE because it benefits from continuity of values at the low sampling interval. AKE and DESM both are based on spatial neighbors’ correlation information, so they have lower RMSE. Compared with them, TSNN can use time distance and temporal neighbors’ correlation information as well, which make it get the lowest RMSE. Among all algorithms, TSNN has the lowest RMSE.

Figure 8 shows experimental results for missing humidity measuring data. Similarly, it shows that the RMSE for all imputation algorithms fluctuates as the percent of missing values increases, LIN has a notable growing trend because the temporal neighbors will become lesser with the increasing percent of missing values, and the humidity data have more dramatic fluctuation than temperature data. In comparison, using data distance rather than time distance, TKNN gets lower RMSE in this situation. TSNN still has the lowest RMSE because it can use both data distance and the temporal neighbors’ correlation information. In addition, RMSE for missing humidity data is higher than that for missing temperature data because the humidity measuring data have more dramatic variety than temperature measuring data, which is due to more affected factors in the physical environment. 

Figure 9 demonstrates the RMSE for all imputation algorithms for missing temperature measurement data (a) and humidity measurement data (b) in the different data density. With the increasing sampling interval, among algorithms without correlation information, SKNN has less fluctuate because it has no connection with temporal neighbors, which will be affected by sampling interval significantly, while the RMSE of LIN and TKNN go up sharply because temporal neighbors will become lesser. In contrast, the correlation-based algorithms, AKE, DESM and TSNN, have better performance. AKE has lower RMSE than DESM because of more weight-based spatial neighbors’ contributions, but TSNN will obtain more benefits from values of spatial neighbors in geometrical and data distance when the sampling interval is increasing, which makes it have the lowest RMSE among all algorithms.

**Figure 10:**
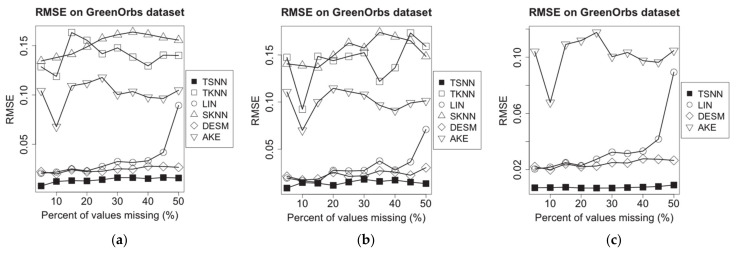
RMSE of imputation algorithms on temperature data of GreenOrbs dataset for the three scenarios: (**a**) temperature NA and humidity not NA, (**b**) temperature NA and humidity 50% NA and (**c**) temperature NA and humidity NA.

**Figure 11:**
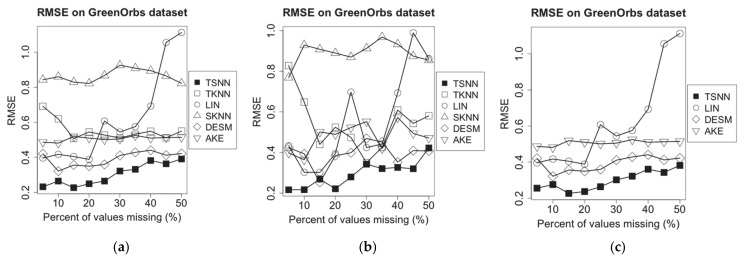
RMSE of imputation algorithms on humidity data of GreenOrbs dataset for the three scenarios: (**a**) temperature NA and humidity not NA, (**b**) temperature NA and humidity 50% NA and (**c**) temperature NA and humidity NA.

On GreenOrbs dataset, for the missed temperature measurement data, we set the sampling interval as 1.5 min and missing percentages range as from 5% to 50%; then, we make the same experiments on the three test scenarios, and the results are shown in Figure 10. Similarly, we get experimental results for the missing humidity measurement data shown in Figure 11.

**Figure 12:**
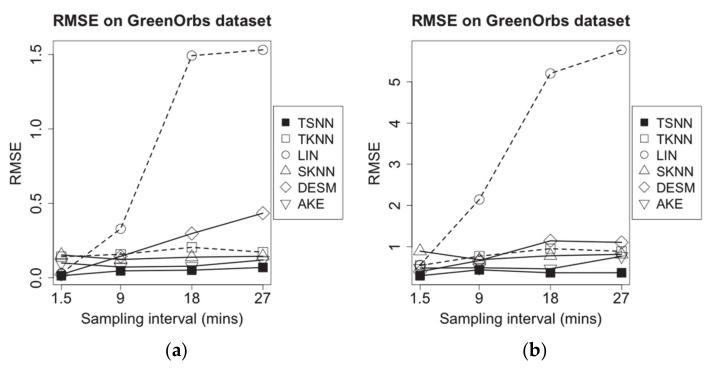
RMSE of imputation algorithms on different data density for GreenOrbs dataset: (**a**) temperature data and (**b**) humidity data.

Next, we set sampling intervals from 1.5 min to 27 min, to make the new test datasets and repeat the same experiments for evaluating the performance of algorithms on different data density. The results are shown as Figure 12.

From Figure 10 and Figure 11, we find that in GreenOrbs dataset, both data distance-based algorithms, TKNN and SKNN, have the relative low performance in RMSE because the relationship between the temperature and the humidity data has been affected by more complex environment factors in the outdoor woods than the indoor room. Meanwhile, the temperature and the humidity data in the outdoor woods have more intensive change than that in the indoor room; that causes the time distance-based algorithm, LIN, get worse performance as the percent of missing values increases. In these situations, TSNN algorithm can optimally utilize four kinds of nearest neighbor values in geometrical or time distance and data distance and make effective estimation based on more information, so it still gets the lowest RMSE among all algorithms. 

Moreover, as Figure 12 shows, among all algorithms, the dramatic changes of temperature and humidity measuring data make the performance of LIN, which is only based on time distances, reduce sharply with increasing sampling interval. SKNN and AKE, which are based on spatial nearest neighbor values, get the better performance. Benefitting from both the temporal and spatial nearest neighbor values, TSNN maintains the best performance in RMSE.

The above experimental results on two different test datasets show that TSNN has the best RMSE performance.

## 5. Discussion

The basic idea to make imputation for missing data on nodes of WSNs is to estimate the missing data using the information of non-missing data. Therefore, we can improve PCE with less information during imputation. It is a flexible way to make imputation by combining spatial and temporal nearest neighbor values because the algorithm is still applicable when one of them, spatial or temporal information, is unavailable for some reason. For example, when there are not spatial neighbors, the AKE is unusable, but the TSNN may be still available if it can find temporal neighbors.

On the contrary, to improve RMSE of imputation, we need to maximize the available information, in other words, more information of non-missing data will be helpful to improve the accuracy of estimation. Both of the spatial and temporal information are utilized in the TSNN. Actually, it is not a brand-new way to make imputation using both of them. For example, in DESM, spatial and temporal information are both used as well. However, the crucial point in TSNN is to make the calculated contribution ratio of spatial and temporal information to the estimation approach the actual contribution ratio in the real case. Different from the contribution ratio calculated on the basis of the correlation between the node with missing value and its neighbors in other algorithms, for example, DESM. We define the spatial–temporal coefficient λ, which makes it more accurate to evaluate the actual contribution of spatial and temporal information of non-missing data. It is obtained based on the subset of non-missing information that is chosen randomly in the observed time window W, while the regression tool and average root mean square error is applied in the calculation. The coefficient λ is more reasonable for evaluation the contribution ratio, but it has its own downside: It is costly to compute. Generally, the data processing center of WSNs is able to support this computation but how to optimize the method to get this coefficient and alleviate the cost of computation will be considered in our further research work.

In addition, the correlations among more than one sensor on the same node are utilized in TSNN. They have been described from the perspective of data distances in time and space dimensions. In our algorithm, k-nearest neighbors method is applied in the calculation in which the Gaussian kernel function is used to work out the weight of neighbors. The amounts of spatial and temporal nearest neighbors, $k_{s}$ and $k_{t},$ are key parameters because more neighbors may bring noise whereas less neighbors will provide insufficient information. TSNN can find the best number of neighbors by calculating the correlations among measurements of the node with missing values and its neighbors on the subset without missing values. However, the range of the neighbors still requires being set by users because it is strongly related to the spatial distribution of the nodes in WSNs, especially in the situation that the nodes are deployed in a building with complex structure. In addition, the change of the node’s state is another problem in building the group of neighbors; because of power failure or other reasons, the nodes which are in the previous initial neighbors group may have problems and become unavailable. One way to solve the problem is to maintain the topological structure of all the neighbor groups and update them regularly, but the task will consume lots of computational resources. How to optimize the initial selection of neighbors and reduce the computation is another research objective for the next stage.

## 6. Conclusions

In this paper, we present TSNN, a new algorithm for imputation of missing values in WSNs. As the basis of the algorithm, the temporal and spatial nearest neighbor values have been defined. The missing values of the node have been estimated by utilizing the non-missing values of the nodes in the observed time window, in which the regression tool is applied, and the best number of nearest neighbors and the spatial–temporal coefficient are figured out to make the algorithm obtain its best performance. Two evaluation methods, PCE and RMSE, are applied to evaluate the performance of the algorithms, and we test our algorithm on two WSNs dataset, the indoor dataset Intel LAB and the outdoor dataset GreenOrbs, and compare it with other typical algorithms. The performance study shows that TSNN is able to exploit the spatial and temporal information and impute missing sensor data with higher imputation accuracy and reduce the number of cases that cannot be imputed as well.

## Data Availability

Data sharing not applicable.
